# Clinicopathological Correlates of γδ T Cell Infiltration in Triple-Negative Breast Cancer

**DOI:** 10.3390/cancers13040765

**Published:** 2021-02-12

**Authors:** Florence Boissière-Michot, Ghita Chabab, Caroline Mollevi, Séverine Guiu, Evelyne Lopez-Crapez, Jeanne Ramos, Nathalie Bonnefoy, Virginie Lafont, William Jacot

**Affiliations:** 1 Institut Régional du Cancer de Montpellier (ICM), Val d’Aurelle, 34298 Montpellier, France; ghita.chabab@inserm.fr (G.C.); Caroline.Mollevi@icm.unicancer.fr (C.M.); Severine.Guiu@icm.unicancer.fr (S.G.); Evelyne.Crapez@icm.unicancer.fr (E.L.-C.); Jeanne.Ramos@icm.unicancer.fr (J.R.); nathalie.bonnefoy@inserm.fr (N.B.); virginie.lafont@inserm.fr (V.L.); William.Jacot@icm.unicancer.fr (W.J.); 2 Institut de Recherche en Cancérologie de Montpellier (IRCM), Inserm U1194, 34298 Montpellier, France; 3 Montpellier University, 34090 Montpellier, France

**Keywords:** triple-negative breast cancer, γδ T cells, prognosis, basal-like, *BRCA1*, *PIK3CA*

## Abstract

**Simple Summary:**

The prognostic impact of the different tumor-infiltrating lymphocyte (TIL) subpopulations remains debated in solid cancers. We investigated the clinicopathological correlates and prognostic impact of TILs, particularly of γδ T cells, in 162 triple-negative breast cancer (TNBC) patients. A high γδ T cell density was significantly associated with younger age, higher tumor histological grade, adjuvant chemotherapy, *BRCA1* promoter methylation, TIL density, and PD-L1 and PD-1 expression. In multivariate analyses, γδ T cell infiltration was an independent prognostic factor. However, this prognostic impact varied according to the tumor *PIK3CA* mutational status. High γδ T cell infiltration was associated with better survival in patients with *PIK3CA* wild-type tumors, without significant difference in the *PIK3CA*-mutated tumor subgroup. Altogether, these data suggest that high γδ T cell infiltrate is correlated with immune infiltration and might represent a prognostic tool in TNBC patients.

**Abstract:**

The prognostic impact of the different tumor-infiltrating lymphocyte (TIL) subpopulations in solid cancers is still debated. Here, we investigated the clinicopathological correlates and prognostic impact of TILs, particularly of γδ T cells, in 162 patients with triple-negative breast cancer (TNBC). A high γδ T cell density (>6.625 γδ T cells/mm^2^) was associated with younger age (*p* = 0.008), higher tumor histological grade (*p* = 0.002), adjuvant chemotherapy (*p* = 0.010), *BRCA1* promoter methylation (*p* = 0.010), TIL density (*p* < 0.001), and PD-L1 (*p* < 0.001) and PD-1 expression (*p* = 0.040). In multivariate analyses, γδ T cell infiltration (cutoff = 6.625 γδ T cells/mm^2^) was an independent prognostic factor (5-year relapse-free survival: 63.3% vs. 89.8%, *p* = 0.027; 5-year overall survival: 73.8% vs. 89.9%, *p* = 0.031, for low vs. high infiltration). This prognostic impact varied according to the tumor *PIK3CA* mutational status. High γδ T cell infiltration was associated with better survival in patients with *PIK3CA* wild-type tumors, but the difference was not significant in the subgroup with *PIK3CA*-mutated tumors. Altogether, these data suggest that high γδ T cell infiltrate is correlated with immune infiltration and might represent a candidate prognostic tool in patients with TNBC.

## 1. Introduction

Triple-negative breast cancers (TNBCs) represent 15% of all breast cancers (BCs) and are defined by the absence of estrogen receptors (ERs), progesterone receptors (PRs), and HER2 overexpression/amplification [1,2]. Despite good chemosensitivity of TNBC, its prognosis is poor [1,2,3]. Tremendous efforts have been made in the last decades to better characterize TNBC subtypes. Based on hierarchical clustering, Perou et al. initially described five intrinsic molecular BC subtypes, including a basal-like subtype [4,5]. TNBCs represent approximately 70% of all basal-like BCs, and 80% of the TNBCs can be classified as basal-like tumors using the PAM50 classifier [6]. Immunohistochemistry (IHC) with an expanded surrogate panel of markers (ER, PR, HER2, EGFR, and cytokeratin (CK) 5/6) has provided a more specific definition of basal-like BC [7]. Finally, Lehmann et al. classified a set of 587 TNBC into six subtypes with unique gene expression profiles and ontologies: luminal androgen receptor (AR), basal-like 1 and 2, immunomodulatory, mesenchymal, and mesenchymal stem-like [8]. These subtypes have different therapeutic targets, chemosensitivity levels, and stromal characteristics [9,10].

It is also described that genetic alterations in tumor cells influence the tumor microenvironment. The PIK3CA pathway is the most common activated pathway in breast cancer (for a review, see [11]). In ER+ breast cancers, Sobral-Leite et al. have shown that PIK3CA pathway alteration was associated with CD8 infiltration [12]. In a mouse model of invasive lobular carcinoma of the breast, activation of the PIK3CA pathway led to immune suppression and exhaustion [13]. In TNBC, we previously showed that exon 9 *PIK3CA* mutation was an independent poor prognostic factor [14].

Besides the molecular features, the immune infiltrate also varies in the different BC subtypes. TNBCs show higher density of tumor-infiltrating lymphocytes (TILs) than other BC subtypes, probably because of their higher number of antigenic tumor variants, neoepitope load, and tumor mutational burden [15]. In TNBC, stromal TILs are considered a strong prognostic factor and patients with a high TIL density show better survival [16,17,18,19]. Guidelines for the reliable and reproducible scoring of TIL density have been issued [20] for the routine management of primary BC, in addition to other prognostic markers.

The tumor immune microenvironment comprises heterogeneous populations of different lymphocyte subtypes, predominantly T cells and then B cells, natural killer (NK) cells, macrophages, and dendritic cells (DCs) [21]. The tumor immune cell infiltration differs among TNBC subtypes. This suggests that the immune response can be modulated by the cancer subtype. It also underlies the complex cross-talk between cancer cells and the immune microenvironment [10,22] and its critical role in the cancer outcome. However, in breast cancer, besides the global evaluation of stromal TIL density, there is no consensus to date on the clinical relevance of analyzing the extent of tumor infiltration by specific immune populations [20,23,24]. Particularly, the prognostic value of tumor infiltration by lymphocyte subpopulations, such as different T helper CD4^+^ cell subsets (Th1, Th2, Th17, and FOXP3^+^ regulatory T cells), B cells, cytotoxic NK cells, γδ T cells, and myeloid cells, is poorly documented. Although IHC-based subtyping could improve accuracy, it does not seem to add any new information for outcome prediction compared with their morphology [20]. Therefore, it is important to better describe the TNBC immune microenvironment to precisely understand the mechanisms driving the immune-regulatory processes. This might allow improving TNBC clinical management and developing new therapeutic strategies.

In this context, a recent study emphasized the importance of investigating the role of γδ T cell populations in TNBC outcome [25]. Indeed, Wu et al. demonstrated that progression-free survival and overall survival (OS) correlate with the density of Vδ1^+^ T cells, a subset of γδ T cells. γδ T cells belong to the family of non-conventional or innate lymphocytes that display both T cell and NK cell characteristics (T cell receptor (TCR), NK receptor, Fc receptor expression, etc.). Two main γδ T cell subtypes are present in humans: Vδ1 T cells, mainly found in tissues, and Vγ9Vδ2 T cells, mainly found in peripheral blood. Both subsets have been detected in the microenvironment of solid tumors (e.g., melanoma, breast, colon, lung, ovary, and prostate), and several studies have shown that they participate in the immune response against many solid and hematological malignancies [26,27,28,29,30]. γδ T cells unveil their anti-tumor activities by displaying direct cytolytic activities against transformed cells or/and by stimulating or regulating the biological functions of other immune cells, such as DCs, interferon-γ-producing CD8 αβ T cells, and NK cells [27,28,29]. γδ T cells are considered as attractive therapeutic targets for anti-tumor immunotherapies because of their unique properties. Indeed, they display a strong major histocompatibility complex-independent reactivity against many tumor cell types; they also have no alloreactivity and can be massively expanded from human samples [31]. Although evidence indicates that γδ T cells have a role in cancer, data on their frequency in cancer tissues and clinicopathological correlates remain scarce, particularly in TNBC, for which only a small number of samples have been analyzed [25,32,33,34]. Yet, the precise characterization of TNBC stromal components could allow refining the prognostic evaluation and identifying additional targets for immune modulation in this cancer subtype, for which treatments were lacking.

Here, we investigated the clinicopathological correlates and prognostic impact of TILs, and particularly γδ T cell infiltration, in 162 patients with TNBC. The clinicopathological variables of this cohort, including *PIK3CA* mutation status, were previously described [14,35,36].

## 2. Results

### 2.1. Patient and Tumor Characteristics

**Figure 1:**
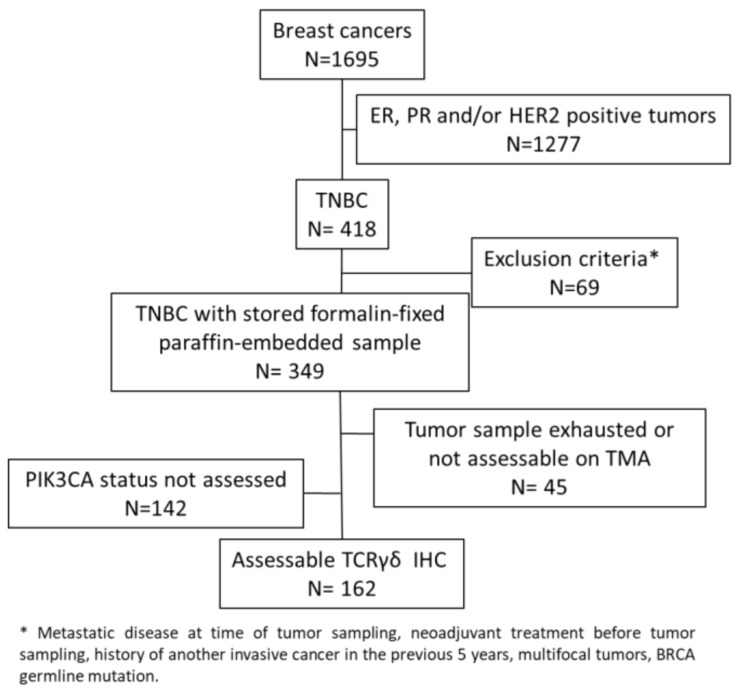
Consort diagram.

In this study, we evaluated γδ T cell infiltration in 162 TNBC samples from chemotherapy-naive patients that were previously analyzed in tissue microarrays (TMAs) (Figure 1) [35,36].

The main clinicopathological characteristics of this cohort of patients with TNBC (Table 1) were consistent with the classical TNBC features. The patients’ median age was 55.4 years (range: 28.7–86.3 years). The most common histological type was ductal carcinoma (82.7%), and 72.0% of the patients received adjuvant chemotherapy. IHC data indicated that 60.6% of the tumors had a basal-like phenotype (i.e., a TNBC tumor that expresses at least one of the two basal markers, EGFR and CK5/6) and 36.5% of the tumors had a molecular apocrine phenotype (i.e., a TNBC tumor that expresses both AR and FOXA1). Moreover, 22.5% of the tumors exhibited *BRCA1* promoter hypermethylation and 13.6% carried a *PIK3CA* mutation (exon 9 or 20).

### 2.2. Association of Tumor-Infiltrating Lymphocytes with TNBC Biological Features

First, we evaluated the association between the immune microenvironment and biological variables in our TNBC cohort (Table S1). The molecular apocrine phenotype was associated with low TIL infiltration (*p* = 0.01), confirmed by the lower CD3^+^ cell density in this TNBC subtype (*p* = 0.024) and with negative PD-L1_TC_ (tumor cells) status (*p* = 0.001). The immune infiltrate, based on TIL, CD3^+^, and CD8^+^ cell quantification, was similar in basal-like and non-basal-like tumors (*p* = 0.353, 0.509, and 0.668, respectively). However, positive PD-L1_TC_ status was associated with the basal-like phenotype (*p* = 0.013) and with *BRCA1* promoter methylation (*p* = 0.030). In TNBC samples with *BRCA1* promoter methylation, the fraction of PD-1-positive cells was higher (*p* = 0.041) and a trend was observed with higher CD3^+^ cell density (*p* = 0.07). *PIK3CA*-mutated tumors were characterized by lower CD8^+^ cell infiltration (*p* = 0.038) and negative PD-L1_SC_ (stromal cells) status (*p* = 0.048). Conversely, tumors with wild-type *PIK3CA* tended to have positive PD-L1_TC_ status (*p* = 0.064). 

Altogether, these results suggest an association between the immune network and the TNBC molecular subtype. Overall, basal-like tumors, which frequently harbor *BRCA1* promoter hypermethylation (in our sample, 80.6% of TNBCs with *BRCA1* promoter hypermethylation were basal-like tumors; *p* = 0.004), were more often associated with positive PD-L1_TC_ and/or PD-1_SC_ status. Conversely, molecular apocrine tumors, which frequently harbor *PIK3CA* mutations (77.3% of *PIK3CA*-mutated tumors had this phenotype in our study; *p* < 0.001), were more often associated with a low TIL density and the absence of PD-L1 and PD-1 expression.

### 2.3. In Situ γδ T Cell Infiltration Analysis

**Figure 2:**
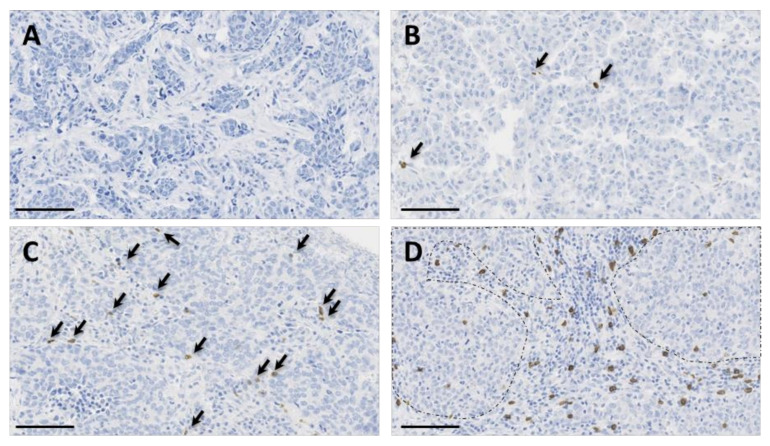
Immunohistochemical staining of γδ T cells in four triple-negative breast cancer (TNBC) samples showing variable γδ T cell infiltration, from absent (panel (**A**)) to high (panel (**D**)) infiltration. γδ T cells are highlighted by arrows in panels (**B**,**C**). In panel (**D**), note the presence of γδ T cells in the inflammatory stroma and inside tumor nests (surrounded by dotted lines). Tissue sections were analyzed by immunohistochemistry (IHC) with an anti-T cell receptor (TCR) δ-chain antibody to detect γδ T cells (brown) and counterstained with hematoxylin. Scale bar: 100 µm.

We used the recently validated monoclonal antibody H-41 [37] to detect the TCR δ-chain by IHC in the 162 TNBCs arrayed on TMAs. In TNBC samples, γδ T cell infiltration was very variable (from 0 to 193.8 γδ T cells/mm^2^; median: 6.625). Overall, we observed γδ T cell infiltration in 84.6% of the samples (137/162 TNBC samples had at least one γδ T cell). When present, γδ T cells were mostly located in the stroma and, to a lower extent, inside tumor nests (Figure 2).

### 2.4. Association of In Situ γδ T Cell Infiltration with Clinicopathological Features

To determine the clinical significance of γδ T cells in TNBC, we analyzed the main clinicopathological features relative to the γδ T cell infiltration level (Table 2). A high γδ T cell density (above the median value of 6.625 cells/mm^2^) was associated with younger age (<55 years; *p* = 0.008); higher histological grade (*p* = 0.002); adjuvant chemotherapy (*p* = 0.010); *BRCA1* promoter methylation (*p* = 0.010); TIL, CD3^+^, and CD8^+^ cell infiltration (*p* < 0.001 for all three parameters); PD-L1 expression by tumor cells (*p* < 0.001) and stromal cells (*p* < 0.001); and PD-1 expression (*p* = 0.040). Conversely, γδ T cell infiltration was not correlated with tumor size, nodal status, histological type, basal-like phenotype, *PIK3CA1* mutations, and AR or FOXA1 expression. Nevertheless, we observed a trend between low γδ T cell density and molecular apocrine phenotype (AR and FOXA1 expression; *p* = 0.072).

**Figure 3:**
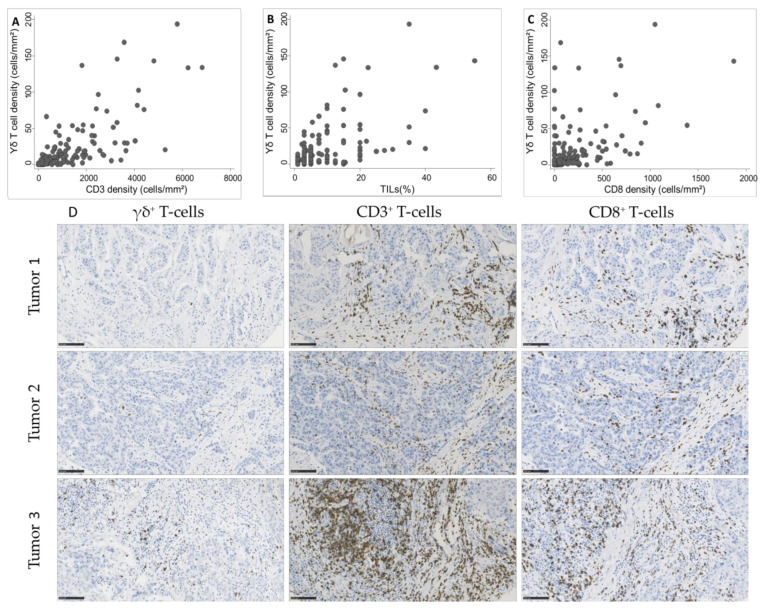
Correlation between γδ T cell density and (**A**) CD3^+^ cell density (Spearman’s rho = 0.74), (**B**) tumor-infiltrating lymphocyte (TIL) infiltration (Spearman’s rho = 0.63), and (**C**) CD8^+^ cell density (Spearman’s rho = 0.51) in 162 TNBC samples. (**D**) Representative images of TNBC with variable γδ T cell densities and corresponding CD3 and CD8 infiltration. Scale bar: 100 µm.

As we observed a significant association between γδ T cell density and TIL infiltration, assessed according to the published guidelines [20] or by CD3^+^ and CD8^+^ cell immunostaining and quantification, we asked whether γδ T cell density was positively correlated with TILs. We observed a strong correlation of γδ T cell density with CD3^+^ cell and TIL density (Spearman’s rho = 0.74 and 0.63, respectively; Figure 3A,B) and a moderate correlation with CD8^+^ cell density (Spearman’s rho = 0.51; Figure 3C). 

### 2.5. Survival Analyses

Using 24 October 2016 as the cutoff date, the median follow-up was 10.4 years (95% CI (9.1–11.5)). During this period, 51 patients died (5-year OS: 81.8%; 95% CI (74.9–87.0)) and 39 had a tumor relapse (5-year relapse-free survival (RFS): 77.1%; 95% CI (69.4–83.1)). The relapse pattern was consistent with the previously reported temporal distribution of relapse risk in patients with TNBC [2,38].

**Figure 4:**
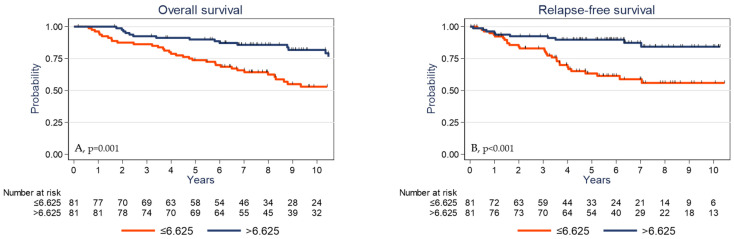
Overall survival (**A**) and relapse-free survival (**B**) as a function of the tumor γδ T cell density (median value used as the cutoff) in the whole cohort of patients with TNBC.

Univariate analysis (Table 3) showed that high pT and pN stages, absence of adjuvant chemotherapy, low TIL and CD3^+^ cell densities, low PD-L1 expression by stromal cells, and low γδ T cell density were significantly associated with shorter OS and RFS. The 5-year OS rates were 73.8% (95% CI (62.6–82.0)) and 89.9% (95% CI (80.9–94.8)) (*p* = 0.001) (Figure 4A) and the 5-year RFS rates were 63.3% (95% CI (50.8–73.5)) and 89.8% (95% CI (80.6–94.8)) (*p* <0.001) in the subgroups with low γδ T cell density and high γδ T cell density, respectively (Figure 4B). OS was significantly associated with younger age (*p* = 0.030) and showed a trend for *PIK3CA* mutations (*p* = 0.061). High tumor infiltration by CD8^+^ cells was associated with longer RFS (*p* = 0.045).

We also analyzed the γδ T/CD3^+^ and γδ T/CD8^+^ cell ratios because the quantitative balance between these TIL subsets might give insights into γδ T cell functional impact. However, these ratios were not associated with OS and RFS, suggesting that the absolute density of each subpopulation is more important than its relative proportion. 

In multivariate analysis (Table 4), high pT and pN stages and low γδ T cell infiltration were associated with shorter OS and RFS. Conversely, adjuvant chemotherapy and lack of *PIK3CA* mutations were associated with better OS and high tumor infiltration by CD8^+^ cells with longer RFS.

In an exploratory analysis, we evaluated the impact of γδ T cell infiltration on specific TNBC subgroups based on the tumor biological profile.

**Figure 5:**
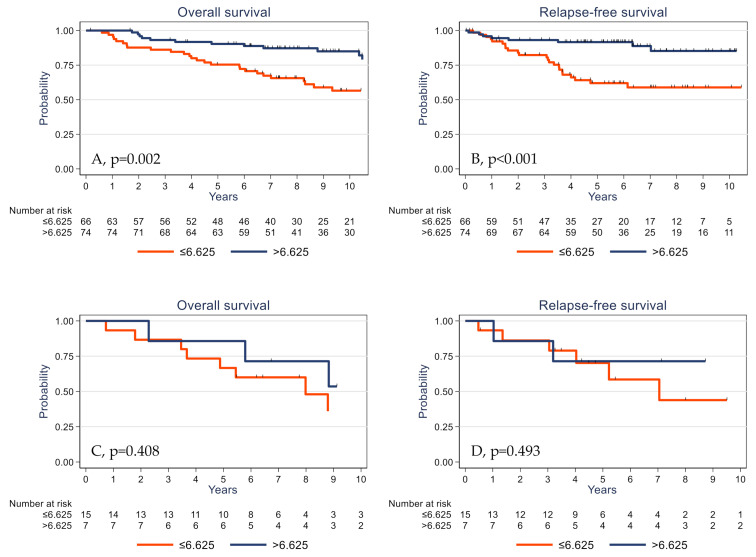
Overall survival (**A**,**C**) and relapse-free survival (**B**,**D**) as a function of the tumor γδ T cell density (median value used as the cutoff) in patients with TNBC harboring wild-type (**A**,**B**) and mutated *PIK3CA* (**C**,**D**).

The prognostic impact of γδ T cell infiltration varied according to the *PIK3CA* mutational status. High γδ T cell infiltration was associated with better survival in *PIK3CA* wild-type tumors (Figure 5A,B) but not in the subgroup of tumors with mutated *PIK3CA* (Figure 5C,D).

To further address the question of the impact of the methylation status of γδ T cells prognosis value, we analyzed OS and RFS in patients with TNBC harboring methylated or unmethylated *BRCA1* promoter. Survival analyses showed a significant prognosis value of γδ T cells regardless of the methylation status of the *BRCA1* promoter.

**Figure 6:**
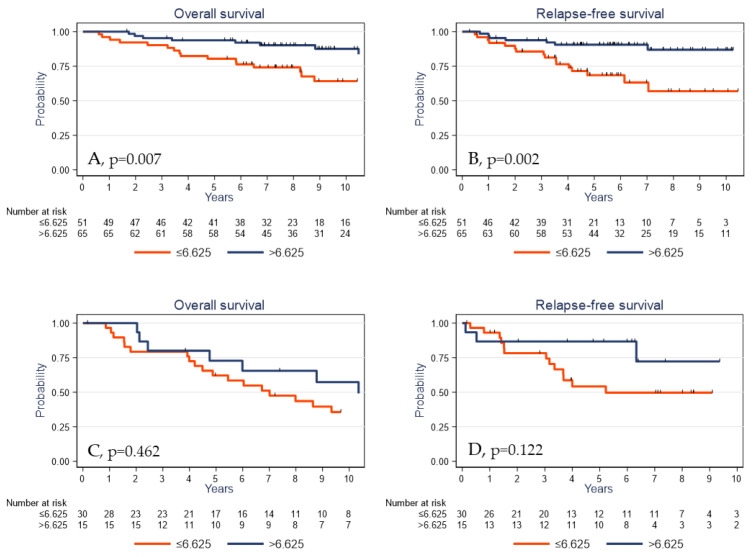
Overall survival (**A**,**C**) and relapse-free survival (**B**,**D**) as a function of the tumor γδ T cell density (median value used as the cutoff) in patients with TNBC treated (**A**,**B**) or untreated (**C**,**D**) with adjuvant chemotherapy.

Finally, to evaluate the predictive value of γδ T cells on clinical outcome under adjuvant treatment, we analyzed the subgroup of patients who received adjuvant chemotherapy. In this subpopulation (*n* = 116), a high γδ T cell density was significantly associated with better OS and RFS (*p* = 0.007 and *p* = 0.002, respectively; Figure 6A,B). Conversely, the γδ T cell density assessed in the untreated TNBC cohort was not significantly associated with OS and RFS (*p* = 0.462 and *p* = 0.122, respectively; Figure 6C,D). Altogether, these data suggest that the prognostic impact of the γδ T cell infiltrate is restricted to the population of patients exposed to adjuvant chemotherapy, possibly due to the immunological effects described with this treatment. However, these latter analyses were carried out on a small number of samples (*n* = 45) and would need to be validated using independent cohorts with larger numbers of samples.

## 3. Discussion

Recently, considerable effort has been focused on targeting immune checkpoints in combination with chemotherapy for TNBC treatment, but with controversial results [39,40,41]. The effect of immunotherapy appears to be linked to tumor mutational burden and genome instability [42] and the functional aspects of immune cell infiltration [43]. As in TNBC, immune cell infiltration differs as a function of their molecular subtypes [21,22], a better description of the immune microenvironment could allow one to precisely determine their prognostic impact and therapeutic opportunities.

Several studies based on the CIBERSORT analysis have reported that intra-tumoral γδ T cell signatures emerged as the most significant favorable cancer-wide prognostic population [44], particularly in all BC types [45]. However, Tosolini et al. [46] demonstrated that the CIBERSORT analysis of pure γδ T cell transcriptome data fails to correctly differentiate these cells from αβ T and NK cells. As we previously validated a γδ T cell detection method by IHC in various human tumors, we used this approach to investigate γδ T cell infiltrate in TNBC [47]. We report here a comprehensive evaluation of the association of immune cell infiltration, particularly γδ T cells, with clinicopathological characteristics and its prognostic impact on 162 patients with TNBC and long-term follow-up.

First, we found, consistently with previous publications [22], that the basal-like phenotype, which is characterized by EGFR and/or CK5/6 expression, frequently related to genomic instability (highlighted in our series by the frequent hypermethylation of the *BRCA1* promoter), was associated with positive PD-L1_TC_ and/or PD-1_SC_ status. Conversely, molecular apocrine tumors, defined as AR- and FOXA1-expressing tumors that frequently harbor *PIK3CA* mutations [48,49], were more frequently associated with a “cold tumor” phenotype (i.e., low TIL density and absence of PD-L1 expression). Altogether, these data reveal important differences between TNBC subtypes and their immune microenvironments.

TILs include different cell populations, but mostly T cells. We previously showed that γδ T cell infiltration tends to be higher in TNBC than in other BC types [47]. Here, we found that in TNBC, a high γδ T cell density was associated with aggressive clinicopathological variables, such as younger age, high histological grade, *BRCA1* promoter hypermethylation, and adjuvant chemotherapy. A high γδ T cell density was also correlated with higher TIL, CD3^+^, and CD8^+^ cell densities and with expression of the PD-1 and PD-L1 immune checkpoint proteins. Overall, these results delineate, to our knowledge for the first time in a large TNBC population, the correlation between γδ T cell density, immune infiltrate extent, and immune exhaustion.

Interestingly, despite its association with pejorative clinicopathological factors, high γδ T cell infiltration was an independent determinant of longer survival (RFS and OS), together with classical clinicopathological prognostic factors, in this population. Moreover, the γδ T cell prognostic value was independent of, or stronger than, the TIL prognostic impact because TILs were not retained in the final multivariate model, highlighting, for the first time, the specific prognostic impact of γδ T cells. 

Several studies have brought evidence of the presence of γδ T cells in the tumor microenvironment of TNBC [25,32,33,34]. Ma et al. previously evaluated the correlations between γδ T cell infiltration and BC characteristics and prognosis in 46 patients [50]. They found a correlation between γδ T cell infiltration, T and N stage, HER2 expression, and worse RFS and OS. In multivariate analysis, γδ T cell infiltration was the most significant independent prognostic factor. However, these 46 patients represented all BC complexity (biology, prognosis, and treatment) and no information was available on TNBC. On the other side, one study by Hidalgo et al. [32] described the localization of γδ T cells in a small cohort of 26 TNBC but did not analyze their correlation with clinical outcome. More recently, Wu et al. [25] and Janssen et al. [34] investigated the role of γδ T cells in TNBC by analyzing their biological functions. Wu et al. showed that γδ TILs from TNBC display a Th1 profile with cytolytic functions and IFN-γ production and their density positively correlates with progression-free and overall survival. Janssen et al. also demonstrated that γδ TILs produce IFN-γ and TNF-α but are not the source of IL-17 in TNBC, contrarily to γδ TILs from colorectal cancer.

The role of the various γδ T cell subsets (Vδ1 and Vγ9Vδ2 T cells) in the tumor microenvironment remains to be clarified. Conversely to Wu et al. [25], who identified Vδ1 T cells as the major human-breast-resident γδ subset, Janssen et al. [34] described Vδ2 T cells as the main subpopulation of the γδ T cells present in TNBC. The small number of patient samples in these two studies (*n* = 11 for each studies) might explain these contradictory results. Moreover, the function of Vδ1 cells in BC is still a matter of debate. We and others [51,52] described potent immunosuppressive functions. Our large population of 162 well-characterized TNBCs allowed us to refine these clinicopathological correlates by validating the results with patient survival, in particular in the patients treated with adjuvant chemotherapy. Together with the biological functions described by Wu et al. and Janssen et al., our data suggest that, in TNBC, γδ T cells display anti-tumor functions through a Th1 profile. Thus, γδ T cell density could be a prognostic and predictive marker in TNBC. One limitation of our study is that we assessed the total γδ T cell population, without identifying the specific Vδ1 or Vδ2 subsets. However, to the best of our knowledge, there is currently no validated antibody to differentiate Vδ1 and Vγ9Vδ2 T cells in formalin-fixed paraffin-embedded samples for the analysis of large BC cohorts. 

Beside the phenotypic dichotomy between Vδ1 and Vγ9Vδ2 T cells, their functional properties can also allow the identification of specific γδ T subpopulations. For instance, a population of IL-17-producing γδ T cells with pro-tumor effects has been described in murine breast, ovarian, and hepatocellular cancer models [53,54,55,56], as well as in human colorectal cancer [53]. In human BC, γδ T cell pro-tumor functions have been associated with the induction of DC senescence [52], while in murine and human pancreatic ductal adenocarcinoma, γδ T cells allow tumor progression by inhibiting αβ T cell activation and infiltration via PD-L1 ligation. Overall, these data support the idea that some γδ T cell subsets can be immunosuppressive and favor tumor progression in selected solid tumor types. We recently reported the presence of γδ T cells in BC, particularly in high-grade tumors, and showed that ~20% of tumor-infiltrating γδ T cells express CD73, an enzyme involved in the production of the immunosuppressive molecule adenosine [51]. These cells also produce IL-10, an immunosuppressive cytokine, and IL-8, a chemokine involved in the recruitment of myeloid-derived suppressor cells. Therefore, some γδ T cells localized in the BC microenvironment might display pro-tumor functions that could favor an immunosuppressive microenvironment and ultimately promote tumor growth [51]. Thus, it would be interesting to combine CD73 and γδ TCR analyses to determine the ratio of CD73^−^ (cytotoxic cells) vs. CD73^+^ (immunosuppressive cells) γδ T cells. To use a γδ T cell as a prognostic marker, it is necessary to analyze CD73^−^ γδ T cell density vs. CD73^+^ γδ T cell density according to patient survival. Nevertheless, on the basis of our results on the prognostic value of γδ T cells in TNBC, we can hypothesize that if they exist, immunosuppressive CD73^+^ γδ T cells should be a minority.

The identification, in our cohort, of a possible association between the prognostic impact of γδ T cell infiltration and *PIK3CA* mutational status highlights the complex cross-talk between oncogenic signaling pathways and anti-tumor immunity [57]. This correlation between PI3K/AKT pathway activation and immune modulation has been previously described. Crane et al. reported that the expression of B7-H1, a negative regulator of T cell function, correlates with PI3K activation in breast and prostate cancers [58]. Recently, Borcoman et al. found a correlation between *PIK3CA* mutations and reduced immune infiltration of the tumor stroma in bladder cancer [59]. Moreover, PI3K inhibition has been associated with higher immune cell infiltration. In our series, the presence of *PIK3CA* mutations was not associated with TIL density (*p* = 0.681; Table S1) and with γδ T cell infiltration (*p* = 0.107). Although this last finding was not significant and the group of *PIK3CA*-mutated tumors was quite small (*n* = 22), it suggests possible differences in γδ T cell functions according to *PIK3CA* status. This hypothesis is strengthened by the observation that the prognostic value of γδ T cell infiltration was restricted, in our series, to the tumors without *PIK3CA* mutations.

Lastly, we identified a relation between the use of adjuvant chemotherapy and the prognostic impact of the γδ T cell infiltrate. While needing validation in an independent data set, considering the number of patients and the subgroup nature of the analysis, our results could emphasize the immunological effects reported with chemotherapy, such as cyclophosphamide, used in breast adjuvant chemotherapies [60,61].

Our findings, if validated in an independent series, could allow the development of strategies based on the characterization of the immune infiltrate and TNBC subtype. They might also help to develop immune intervention strategies for basal-like, immune-infiltrated tumors and anti-PI3K agents, possibly associated with immune modulators, to target the cold tumor phenotype of molecular apocrine tumors.

## 4. Materials and Methods

### 4.1. Objectives

The primary objective was the evaluation of the impact of γδ T cell infiltration, assessed by IHC, on OS and RFS in patients with TNBC. Secondary objectives were the evaluation of the association between γδ T cell infiltration and clinicopathological variables and the multivariate evaluation of the impact of these variables on prognosis (RFS and OS).

### 4.2. Patients and Tumor Samples

We used samples collected between 2002 and 2010 in a dedicated breast cancer prospective database (tumor biobank number BB-0033-00059). We selected only samples with less than 10% of RE- and RP-immunoreactive tumor cells and with HER2 0/1+ by immunohistochemistry and/or not amplified by in situ hybridization. Patients who received neoadjuvant treatment, who had metastatic disease at the time of surgery or multifocal tumors, who had a history of another invasive cancer in the previous 5 years, and those with known germline BRCA mutation were excluded from the study. Overall, 162 patients with available/sufficient tumor tissue and with described CK5/6, EGFR, PD-L1, PD-1, AR, and FOXA1 status (by IHC), as well as *BRCA1* promoter methylation and *PIK3CA* status [35,36,62], were available for the study. None of the patients received targeted therapy or any investigational product, and each patient was treated according to our institution guidelines [63]. The clinicopathological characteristics and treatment of the study cohort are summarized in Table 1. The study was reviewed and approved by the Montpellier Cancer Institute Institutional Review Board (ID number ICM-CORT-2018-34). All patients gave their written, informed consent. As part of the study evaluated the prognostic impact of biological markers, this manuscript adheres to the REMARK guidelines.

### 4.3. Tissue Microarray 

TCRδ expression was analyzed in the same TMA blocks that were used in our previous studies [35,36]. Briefly, for each tumor sample, two cores (1 mm in diameter) were sampled from different malignant areas and arrayed in six TMA blocks. Then, 3 or 4 μm thick sections were cut from the TMA blocks. The 4 µm sections were stained with hematoxylin-eosin-saffron (HES).

### 4.4. Immunohistochemistry

The 3 µm TMA sections were placed on microscope slides (Dako/Agilent, Santa Clara, CA, USA) and dried at room temperature overnight before IHC procedure using the Dako Autostainer Link48 platform and the Flex+ visualization system (Dako/Agilent, Santa Clara, CA, USA). Sections were incubated with the recently validated anti-TCRδ mouse monoclonal antibody (clone H-41, Santa Cruz Biotechnologies, Dallas, TX, USA) at 1:150 dilution at room temperature for 30 min [37] or with anti-CD3 (rabbit polyclonal, Ref A0452, 1:200, 20 min, Dako/Agilent, Santa Clara, CA, USA), anti-CD8 (mouse monoclonal, clone 8/144B, ready to use, 20 min, Dako/Agilent, Santa Clara, CA, USA), anti-CK 5/6 (mouse monoclonal, clone 6D5/16 B4, 1:100, 20 min, Dako/Agilent, Santa Clara, CA, USA), anti-EGFR (mouse monoclonal, clone 31G7, 1:50, 20 min, Invitrogen, Carlsbad, CA, USA), anti-PD-L1 (rabbit monoclonal, clone SP142, 1:200, 30 min, Roche Diagnostics, Basel, Switzerland), anti-PD1 (mouse monoclonal, clone MRQ-22, BioSB, Santa Barbara, CA, USA), anti-RA (mouse monoclonal, clone AR441, 1:150, 30 min, Dako/Agilent, Santa Clara, CA, USA), and anti-FOXA1 (goat polyclonal, Ref HNF-3α/β (C-20), 1:200, 20 min, Santa Cruz Biotechnologies, Dallas, TX, USA) antibodies, as previously described [35,36].

The NanoZoomer slide scanner system (Hamamatsu Photonics, Hamamatsu City, Shizuoka Pref., Japan) was used to digitalize the stained TMA sections with a ×20 objective. TCRδ-positive cells were manually identified and counted within the malignant areas on the digitalized slides with the NDP.view 2.7.39 software (Hamamatsu Photonics, Hamamatsu City, Shizuoka Pref., Japan). Data were expressed as the number of TCRγδ-positive cells per mm^2^. CD3^+^ and CD8^+^ cell densities were assessed using the Histolab^®^ Image Analysis software (Microvision, Evry, France), as previously described [64]. TMA sections stained with other antibodies were analyzed independently by two trained observers, both blinded to the clinicopathological characteristics and patient outcomes at the time of scoring, as previously reported [35,36]. Missing TMA cores, those containing fewer than 10 cancer cells, or those demonstrating significant artefacts were not scored. Discordant cases were reviewed and scored by consensus. 

### 4.5. TIL Assessment

A trained pathologist assessed TILs on HES-stained digitalized TMA sections, following the guidelines issued by the International TIL Working Group [20]. As recommended, only stromal TILs were quantified. Intratumor TILs, defined as intraepithelial mononuclear cells within tumor cell nests, were excluded from the TIL assessment. Stromal TILs are reported as the percentage of area occupied by TILs relative to the whole stroma area.

### 4.6. Tissue Processing and DNA Extraction 

Frozen tumor samples were used for DNA extraction with the QIAamp DNA Mini Kit (Qiagen GmbH, Hilden, Germany) following the manufacturer’s instructions, as previously described [65,66].

### 4.7. Molecular Analysis 

*BRCA1* promoter methylation status was analyzed as previously reported [36]. Briefly, DNA methylation patterns at the CpG islands of the *BRCA1* promoter were assessed using the methylation-specific polymerase chain reaction (PCR) assay [35,66,67], which analyzes seven CpG sites located at −37, −29, −21, −19, +16, +19, and +27 relative to the *BRCA1* exon 1A transcription start site. 

*PIK3CA* mutation status was analyzed by the PCR amplification and high-resolution melting method, followed by sequencing of the purified PCR products [14]. Primers were designed to amplify *PIK3CA* fragments that span the two mutation hotspots in exon 9, including the p.E542X and p.E545X mutations, and exon 20, including the hotspot mutations p.H1047X, p.H1048X, and p.G1049X.

### 4.8. Statistical Analysis

Categorical variables were described by the number of observations and the frequency of each modality and compared with Pearson’s chi-square or Fisher’s exact test. Continuous variables were described by the number of observations, the median, the minimum, and the maximum. OS was defined as the time from surgery to death (whatever the cause). Patients alive or lost to follow-up were censored at the date of last news. RFS was defined as the time from surgery to recurrence. Patients alive without recurrence and patients lost to follow-up were censored at the date of documented visit. Patients who died without recurrence were censored at the date of death. The Kaplan–Meier method was used to estimate the survival rates. The comparison of survival distributions was performed using the log–rank test. Hazard ratios (HRs) with a 95% confidence interval (95% CI) were estimated using a Cox proportional hazard model. Statistical analyses were performed with STATA 16.0 (StatCorp, College Station, TX, USA).

## 5. Conclusions

In TNBC, γδ T cell infiltration is correlated with immune infiltration and with a higher level of T-cell exhaustion, as indicated by its association with the expression of the immune checkpoints PD-L1 and PD-1. After adjusting for common clinicopathological factors in TNBC, γδ T cell infiltration remained a significant favorable prognostic factor for OS and RFS, especially in the *PIK3CA* wild-type subgroup of tumors, and thus represents a candidate prognostic tool. Nevertheless, before its broad application, it must be validated in studies that will also characterize the different γδ T cell populations.

## Figures and Tables

**Table 1:** Patient and tumor characteristics.

Variables	*N* = 162	%
Age (years), median [min–max]	55.4	[28.7–86.3]
<55	79	48.8
≥55	83	51.2
Tumor size (pT)		
T1	70	43.2
T2	79	48.8
T3/T4	13	8.0
Nodal status (pN)		
N−	108	66.7
N+	54	33.3
Histological grade (SBR; 3 missing values)		
1–2	50	31.4
3	109	68.6
Histology		
Ductal	134	82.7
Lobular	6	3.7
Other	22	13.6
Adjuvant chemotherapy (1 missing value)		
No	45	28.0
Yes	116	72.0
Basal-like phenotype (2 missing values)		
No	63	39.4
Yes	97	60.6
AR/FOXA1 expression (1% cutoff; 6 missing values)		
AR^+^/FOXA1^−^	11	7.1
AR^+^/FOXA1^+^	57	36.5
AR^−^	88	56.4
*BRCA1* promoter methylation (2 missing values)		
No Yes	124	77.5
	36	22.5
*PIK3CA* mutations		
None	140	86.4
Exon 9	10	6.2
Exon 20	12	7.4
TILs (%; 4 missing values)		
≤10	124	78.5
>10	34	21.5
CD3^+^ cell density (cells/mm^2^), median [min–max] (6 missing values)	658.41	[0.33–6821]
≤658.41	78	50.0
>658.41	78	50.0
CD8^+^ cell density (cells/mm^2^), median [min–max] (5 missing values)	73.81	[0–1870]
≤73.81	79	50.3
>73.81	78	49.7
PD-L1_TC_ (8 missing values)		
<1%	84	54.6
≥1%	70	45.4
PD-L1_SC_ (%; 8 missing values)		
0	29	18.8
[0–10]	68	44.1
[10–50]	34	22.1
>50	23	15.0
PD-1_SC_ (%; 10 missing values)		
0	45	29.6
[0–10]	50	32.9
[10–50]	46	30.3
>50	11	7.2
γδ T cells density (cells/mm^2^), median [min–max]	6.625	[0–193.8]
≤6.625	81	50.0
>6.625	81	50.0

Basal-like phenotype was considered in the case of positive staining for cytokeratin 5/6 and/or EGFR (>10% of tumor cells stained in IHC). SBR: Scarff–Bloom–Richardson grade; AR: androgen receptor; FOXA1: Forkhead box protein A1; TILs: tumor-infiltrating lymphocytes; PD-L1: programmed cell death ligand 1; PD-1: programmed cell death 1; TC: tumor cells; SC: stromal cells.

**Table 2:** Correlations between clinicopathological features and γδ T cell infiltration.

Variables	γδ T Cell Infiltration (/mm^2^)				
	≤6.625		>6.625		*p*-Value
	*n* = 81	%	*n* = 81	%	
Age (years)					0.008
<55	31	38.3	48	59.3	
≥55	50	61.7	33	40.7	
Tumor size (pT)					0.341
T1	32	39.5	38	46.9	
T2/T3/T4	49	60.5	43	53.1	
Nodal status (pN)					0.096
N−	49	60.5	59	72.8	
N+	32	39.5	22	27.2	
Histological grade (SBR)					0.002
1–2	34	43.0	16	20.0	
3	45	57.0	64	80.0	
Histology					0.325
Ductal	65	80.2	69	85.2	
Lobular	5	6.2	1	1.2	
Other	11	13.6	11	13.6	
Adjuvant chemotherapy					0.010
No	30	37.0	15	18.7	
Yes	51	63.0	65	81.3	
Basal-like phenotype					0.184
No	36	44.4	27	34.2	
Yes	45	55.6	52	65.8	
AR/FOXA1 (1% cutoff)					0.072
AR^+^/FOXA1^+^	35	43.2	22	29.3	
Other	46	56.8	53	70.7	
*BRCA1* promoter methylation					0.010
No	68	86.1	56	69.1	
Yes	11	13.9	25	30.9	
*PIK3CA* mutations					0.107
None	66	81.5	74	91.4	
Exon 9/Exon 20	15	18.5	7	8.6	
TILs					<0.001
≤10%	74	92.5	50	64.1	
>10%	6	7.5	28	35.9	
CD3^+^ cell density (cells/mm^2^)					<0.001
≤658.41	65	84.4	13	16.5	
>658.41	12	15.6	66	83.5	
CD8^+^ cell density (cells/mm^2^)					<0.001
≤73.81	54	70.1	25	31.2	
>73.81	23	29.9	55	68.8	
PD-L1_TC_					<0.001
<1%	58	74.4	26	34.2	
≥1%	20	25.6	50	65.8	
PD-L1_SC_					<0.001
≤10%	63	80.8	34	44.7	
>10%	15	19.2	42	55.3	
PD-1_SC_					0.040
≤10%	53	70.7	42	54.5	
>10%	22	29.3	35	45.5	

Basal-like phenotype was considered in the case of positive staining for cytokeratin 5/6 and/or EGFR (>10% of tumor cells stained in IHC). SBR: Scarff–Bloom–Richardson grade; AR: androgen receptor; FOXA1: Forkhead box protein A1; TILs: tumor-infiltrating lymphocytes; PD-L1: programmed cell death ligand 1; PD-1: programmed cell death 1; TC: tumor cells; SC: stromal cells.

**Table 3:** Univariate analysis.

Variables	OS		RFS	
	HR	95% CI	HR	95% CI
Age (years)		*p* = 0.030		*p* = 0.177
<55	1		1	
≥55	1.86	1.05–3.31	1.55	0.81–2.96
Tumor size (pT)		*p* < 0.001		*p* < 0.001
T1	1		1	
T2/T3/T4	3.44	1.76–6.70	4.07	1.80–9.24
Nodal status (pN)		*p* < 0.001		*p* < 0.001
N−	1		1	
N+	2.66	1.54–4.60	5.10	2.62–9.93
Histological grade (SBR)		*p* = 0.966		*p* = 0.526
1–2	1		1	
3	0.99	0.55–1.77	1.25	0.62–2.51
Histology		*p* = 0.448		*p* = 0.442
Ductal	1		1	
Other	0.74	0.33–1.65	0.70	0.27–1.80
Adjuvant chemotherapy		*p* < 0.001		*p* = 0.036
No	1		1	
Yes	0.32	0.19–0.56	0.49	0.26–0.94
Basal-like phenotype		*p* = 0.329		*p* = 0.964
No (≤10%)	1		1	
Yes (Basal)	1.33	0.74–2.37	1.02	0.53–1.94
AR/FOXA1		*p* = 0.501		*p* = 0.272
AR^+^/FOXA1^+^	1		1	
Other	0.83	0.47–1.44	0.70	0.37–1.32
*BRCA1* promoter methylation		*p* = 0.701		*p* = 0.208
No	1		1	
Yes	0.87	0.44–1.75	0.59	0.25–1.41
*PIK3CA* mutations		*p* = 0.061		*p* = 0.202
None	1		1	
Exon 9/Exon 20	1.98	1.01–3.86	1.71	0.78–3.71
TILs		*p* = 0.005		*p* = 0.001
≤10%	1		1	
>10%	0.29	0.10–0.80	0.17	0.04–0.69
CD3^+^ cell density (cells/mm^2^)		*p* = 0.010		*p* < 0.001
≤658.41	1		1	
>658.41	0.48	0.27–0.85	0.26	0.12–0.55
CD8^+^ cell density (cells/mm^2^)		*p* = 0.312		*p* = 0.045
≤73.81	1		1	
>73.81	0.75	0.42–1.32	0.51	0.27–0.99
PD-L1_TC_		*p* = 0.125		*p* = 0.150
<1%	1		1	
≥1%	0.65	0.37–1.14	0.62	0.32–1.20
PD-L1_SC_		*p* = 0.004		*p* = 0.002
≤10%	1		1	
>10%	0.41	0.21–0.78	0.30	1.14–0.69
PD-1_SC_		*p* = 0.403		*p* = 0.808
≤10%	1		1	
>10%	1.27	0.72–2.22	1.08	0.57–2.07
γδ T cell density (cells/mm^2^)		*p* = 0.001		*p* < 0.001
≤6.625	1		1	
>6.625	0.39	0.22–0.70	0.28	0.14–0.58

OS: overall survival; RFS: relapse-free survival; HR: hazard ratio; CI: confidence interval; basal-like phenotype was considered in the case of positive staining for cytokeratin 5/6 and/or EGFR (>10% of tumor cells stained in IHC); SBR: Scarff–Bloom–Richardson grade; AR: androgen receptor; FOXA1: Forkhead box protein A1; TILs: tumor-infiltrating lymphocytes; PD-L1: programmed cell death ligand 1; PD-1: programmed cell death 1; TC: tumor cells; SC: stromal cells.

**Table 4:** Multivariate analysis.

Variables	OS *N* = 161		RFS *N* = 157	
	HR	95% CI	HR	95% CI
Tumor size		*p* = 0.003		*p* = 0.002
T1	1		1	
T2/T3/T4	2.73	1.34–5.56	3.37	1.45–7.83
Nodal status		*p* = 0.002		*p* < 0.001
N−	1		1	
N+	2.64	1.42–4.92	4.39	2.16–8.91
Adjuvant chemotherapy		*p* < 0.001		
No	1			
Yes	0.27	0.14–0.49		
*PIK3CA* mutations		*p* = 0.032		
None	1			
Exon 9/Exon 20	2.25	1.12–4.49		
CD8^+^ cell density (cells/mm^2^)				*p* = 0.017
≤73.81			1	
>73.81			0.43	0.21–0.87
γδ T cell density (cells/mm^2^)		*p* = 0.031		*p* = 0.011
≤6.625	1		1	
>6.625	0.51	0.28–0.96	0.39	0.18–0.84

OS: overall survival; RFS: relapse-free survival; HR: hazard ratio; CI: confidence interval.

## Data Availability

No new data were created or analyzed in this study. Data sharing is not applicable to this article.

## Supplementary Materials

The following is available online at https://www.mdpi.com/2072-6694/13/4/765/s1: Table S1: Associations between immune microenvironment and TNBC biological variables.

## Abbreviations

AR	Androgen receptor
BC	Breast cancer
*BRCA1*	Breast cancer type 1 susceptibility gene
CK 5/6	Cytokeratin 5/6
DC	Dendritic cells
DNA	Deoxyribonucleic acid
EGFR	Epidermal growth factor receptor
ER	Estrogen receptor
FOXA1	Forkhead box protein A1
HES	Hematoxylin-Eosin-Saffron
HER2	Human epidermal growth factor receptor 2
HR	Hazard ratio
IHC	Immunohistochemistry
NK	Natural killer
OS	Overall survival
PCR	Polymerase chain reaction
PD-1	Programmed cell death 1
PD-L1	Programmed cell death ligand 1
PR	Progesterone receptor
REMARK	Reporting recommendations for tumor marker prognostic studies
RFS	Relapse-free survival
SC	Stromal cells
TC	Tumor cells
TCR	T cell receptor
TIL	Tumor infiltrating lymphocyte
TMA	Tissue microarray
TNBC	Triple-negative breast cancer
