# Supplementary material for: Clinicopathological Correlates of γδ T Cell Infiltration in Triple-Negative Breast Cancer
Source: Cancers (Basel). 2021 Feb 12;13(4):765. doi: 10.3390/cancers13040765 (PMC7918092; doi:10.3390/cancers13040765)
Supplement: Supplementary file 1 [file cancers-13-00765-s001.pdf]

Supplementary table 1: Associations between immune microenvironment and TNBC biological variables

	**TILs**					**CD3**					**CD8**				
	**≤ 10**		**> 10**		***p***	**≤ 658.41**		**> 658.41**		***p***	**≤ 73.81**		**> 73.81**		***p***
	n	**%**	***n***	**%**		***n***	**%**	***n***	**%**		***n***	**%**	***n***	**%**	
**AR/FOXA1 (1% cut-off)**					0.010					0.024					0.474
AR$^+$/FOXA1$^+$	51	42.9	6	18.2		34	44.7	20	27.0		30	38.5	24	32.9	
Other	68	57.1	27	81.8		42	55.3	54	73.0		48	61.5	49	67.1	
**Basal-like phenotype**					0.353					0.509					0.668
No	52	42.3	11	33.3		32	41.6	28	36.4		32	41.0	29	37.7	
Yes	71	57.7	22	66.7		45	58.4	49	63.6		46	59.0	48	62.3	
***BRCA1* promoter methylation**					0.224					0.070					0.671
No	98	80.3	24	70.6		63	82.9	55	70.5		58	75.3	61	78.2	
Yes	24	19.7	10	29.4		13	17.1	23	29.5		19	24.7	17	27.8	
***PIK3CA* mutations**					0.681					0.168					0.038
None	106	85.5	30	88.2		64	82.0	70	89.7		64	81.0	72	92.3	
Exon 9/Exon 20	18	14.5	4	11.8		14	18.0	8	10.3		15	19.0	6	7.7	

	**PD-L1$_{TC}$**					**PD-L1$_{SC}$**					**PD-1$_{SC}$**				
	**< 1%**		**≥ 1%**		***p***	**≤ 10**		**> 10**		***p***	**≤ 10**		**> 10**		***P***
	n	**%**	***n***	**%**		***n***	**%**	***n***	**%**		***n***	**%**	***n***	**%**	
**AR/FOXA1 (1% cut-off)**					0.001					0.097					0.351
AR$^+$/FOXA1$^+$	40	48.2	15	22.1		39	41.5	16	28.1		36	39.1	18	31.6	
Other	43	51.8	53	77.9		55	58.5	41	71.9		56	60.9	39	68.4	
**Basal-like phenotype**					0.013					0.107					0.552
No	41	48.8	20	29.0		43	44.8	18	31.6		35	37.2	24	42.1	
Yes	43	51.2	49	71.0		53	55.2	39	68.4		59	62.8	33	57.9	
***BRCA1* promoter methylation**					0.030					0.161					0.041
No	70	84.3	48	69.6		78	81.3	40	71.4		77	82.8	39	68.4	
Yes	13	15.7	21	30.4		18	18.7	16	28.6		16	17.2	18	31.6	
***PIK3CA* mutations**					0.064					0.048					0.284
None	68	81.0	64	91.4		79	81.4	53	93.0		79	83.2	51	89.5	
Exon 9/Exon 20	16	19.0	6	8.6		18	18.6	4	7.0		16	16.8	6	10.5	

Footnote: Basal-like phenotype was considered in the case of positive staining for cytokeratin 5/6 and/or EGFR (>10% of tumor cells stained in IHC); AR: androgen receptor; FOXA1: Forkhead box protein A1; TILs: tumor-infiltrating lymphocytes; PD-L1: programmed cell death ligand 1; PD-1: programmed cell death 1; TC: tumor cells; SC: stromal cells.
